# The molecular population structure of *Swertia perennis* (Gentianaceae) in Central Europe

**DOI:** 10.1038/s41598-023-43731-5

**Published:** 2023-10-10

**Authors:** Jacek Urbaniak, Paweł Kwiatkowski

**Affiliations:** 1 Department of Botany and Plant Ecology, Wrocław University of Environmental and Life Sciences, Wrocław, Poland; 2 Institute of Biology, Biotechnology, and Environmental Protection, University of Silesia in Katowice, Katowice, Poland

**Keywords:** Biodiversity, Biogeography, Conservation biology, Ecosystem ecology

## Abstract

Phylogeographic analysis of *Swertia perennis,* a typical European subalpine springtime species, revealed the existence of at least five major phylogenetic lineages. A large phylogeographic separation exists among these geographical regions, which confirms the existence of glacial refugia in the Pyrenees, but also in the Eastern and Central Alps. The results obtained from the analyzes indicate the existence of three major differences between the populations of the Alps and the Pyrenees, but also between the populations of the Alps and those of other geographical regions (Carpathians, southern Czech Republic, Sudetes and northern Poland). The studied populations from the Black Forest and from north-eastern and southern Poland are a relic of the former wider distribution of these (periglacial) genetic groups within Swertia perennis. Our results also confirm the existence of biogeographical links between the Carpathians and the Hercynian Range and the Alps. Certainly, there was an exchange of genes between populations located in the eastern Alps, the Carpathians and the Hercynian ranges (Czech Republic, Jeseníky, Sudetes, Ore Mountains). This confirms previous results of comparative studies on the genetic diversity of populations of other vascular plant species.

## Introduction

The Quaternary history of the flora of Europe was largely shaped by climatic oscillations during the Pleistocene. (from 2.58 million to 11.7 thousand years ago). This was accompanied by a drop in temperature and an increase in snowfall, which favored the formation of a thick ice cover and decreasing of the temperature^1,2^. As a result, the Pleistocene experienced several glacial phases that strongly influenced the geographic range of many plant and animal species. Difficult climatic conditions and thousands of years of periods of low temperature during the climate cooling and higher temperatures during the interglacials resulted in alternately shrinking and splitting the ranges of species, which often led to the isolation of populations^3^. By contrast, during warmer periods, many populations of species moved back to their original ranges through return migration or recolonization. Under such conditions, the ranges of species expanded, which led to the restoration of gene flows^4–6^. However, genetic changes that occurred in populations isolated during glaciations often led to complex mechanisms of speciation. In refugial populations, long-term isolation can substantially affect genetic diversity and reduce population size^6,7^. Other evolutionary processes may, in turn, take place in a recently colonized area. Some of these processes are the founder effect or the bottleneck effect, which cause a reduction or impoverishment of the gene pool of the population^1,8,9^. In turn, the opposite process that may take place in the contact zones between migrating populations may increase genetic diversity^1,6,10,11^.

Tracking the changes and transformations of flora over thousands of years is difficult because of the very limited research possibilities to study fossil material (palynology) or perform molecular research^12–14^. For this reason, and because of the difficulties resulting from the collection of research material, there is little research that would allow for an in-depth understanding of the processes that occurred in plant species populations during the Pleistocene. Although there are studies on the variability and genetic differentiation of populations, found in the Alps and the Carpathians, studies on the populations of species found in the lowlands and in other mountainous regions are limited. Although there are studies on the variability and genetic differentiation of populations found in the Alps and the Carpathians, studies on the populations of species found in the lowlands but also in the Sudetes, Pyrenees and Dinaric Mountains are not numerous. Each of these mountainous regions has historically played an important role as a refuge for species in Pleistocene Europe^2,10^. In the interglacials and just after the end of the ice age, many plant species migrated in different directions from these mountains, expanding their ranges. These processes took place both from the Apennines and the Pyrenees towards the Alps and the Balkans, from the Alps towards the north (Sudetes) through the Šumava Mountains, but also from the Dinaric Mountains towards the Carpathians and between the Sudetes and the Carpathians^15–17^.

Therefore, here, we present the results of research on the genetic differentiation of the population of a rare species from the Gentianaceae family: *Swertia perennis*, referring to the history of colonization and recolonization during the post glacial period. *S. perennis* is interesting for several reasons. It occurs in a large geographical area both in the European lowlands and at high altitudes in the mountains. *S. perennis* is also a disjunct species, which is widely distributed in Europe, but occurs either in the lowlands (e.g., northern Poland and Germany) or in the mountains (e.g., the Sudetes, the Carpathians, and the Alps). Based on the currently available data, determining the origin of *S. perennis* in the areas where it is found today but also the relationships between the widespread populations of *S. perennis* and the reasons for the discontinuity of its range is difficult. It is possible that the current populations in the Carpathians, the Sudetes, the Alps, the Pyrenees, and the Balkans are populations that survived in glacial refugia, where the plant may have survived the unfavorable glacial period, like many other Arctic-Alpine species^8,18,19^.

Lowland areas in Europe were glaciated the longest, and the last glaciation in Northern Europe (including northern Poland) ended about 10.4 thousand years ago^18–20^. In addition, several species such as *Pulsatilla vernalis, Doronicum austriacum* or *Salix lapponum*, have survived to this day in the currently fragmented European mountain ranges by inhabiting refuges in non-glaciated zones of the mountains and in areas on the outskirts of glaciations^21^. Discovered the main genetic groups of *P. vernalis* in the Alps as well as in the lowlands north of the European Alpine system. This suggests that colonization of the lowlands may have occurred independently, with later zone contact in the German and Polish lowlands. A common haplotype was found both for the population of *D. austriacum* from mountainous areas (Carpathians, Sudetes) and those located in the Świętokrzyska Upland (Poland), several hundred kilometers to the north, surrounded by lowland areas^22^. Similarly, *S. lapponum* is a species that at its current sites, both in the Sudetes and in the lowland of north-eastern Poland, shows a large phylogeographic similarity^23^**.**

Therefore, to fully understand the complexity of the problem resulting from genetic differences that arose in populations during the Pleistocene, particularly in Central Europe, genetic analyses covering the occurrence of the species in a wide distribution range are needed. This applies in particular to the study of species occurring both in the mountains and in lowlands, which are few. Closer phylogeographic relations between mountainous and lowland areas, whose flora developed differently, are not known. Both differences and similarities in halpotypes between such disjunct populations are found. The reason is probably the existence of different biogeographical patterns that shaped the origin of plant populations in the past^15,21^. For this purpose, we chose *S. perennis*, a widespread species found from northern Siberia to the Pyrenees. We collected samples not only in the plant’s meridional distribution (Carpathians–Sudetes–Alps–Pyrenees) but also in its latitudinal distribution (northern Poland–southern Poland–Czech Republic). The goal was to analyze the history of *S. perennis* in the late Pleistocene. Unfortunately, the analyzes performed could not reflect the entire whole biogeographical history of *S. perennis* in the World and were limited only to Europe (from the Pyrenees to the Carpathians). Unfortunately, the conducted analyzes were not able to reflect the entire biogeographical history of *S. perennis* in the world, but they are another contribution to understanding the biogeography of this species in the context of climate changes in the Pleistocene.

The main questions posed in this work were: (1) what level of genetic differentiation *S. perennis* exhibits (2) can genetic phylogeographical groups be distinguished among European populations?

## Material and methods

### The species

*Swertia perennis* Linnaeus, Sp. Pl. 226, 1753, syn.: *Gentiana palustris* All. Fl. Pedem. 1: 100, 1785 belong to the Gentianaceae family inhabiting higher mountains but it can also be found at lower elevations in the lowlands throughout Europe from the Urals to the Pyrenees. The species is distributed globally across the Northern hemisphere, but also shows discontinuous distribution from Asia to North America^23^. It inhabits wetlands, especially peat bogs in calcareous fens or high mountains medows; however, it is also present in, creek shores or wet rocks. It is a species of circum-boreal distribution, inhabiting Arctic, Siberian and Northern, Atlantic and Central European provinces^24,25^. In Europe, the species can be found in all mountain range systems: in Pyrenees, Alps, Carpathians, Herzynian mountains (Sudetes, Bohemian Forest, Erzgebirge), Caucasus and Dinaric Alps. *S. perennis* is a long-lived perennial rhizome herb that usually produces one erect stem that grows to around 10–50 cm tall. It is a diploid (2*n* = 28) organism that flourishes in July or August^24,25^. The flower nectar chambers are visited by various insects including species of Coleoptera, Lepidoptera, Diptera and Hymenoptera (especially *Bombus* and *Vespidae*)^24^.

### The species sampling

Plant material of *S. perennis* was collected across all of Europe (Poland, Slovakia, Czech Republic, Germany, Austria, Switzerland, France, and Spain) and preserved in silica gel prior to analysis (Table 1). Eight to ten samples from different geographic locations (Eastern Poland, Northern Poland, Sudetes, Carpathians, Šumava Mountains, Alps, Jura and Pyrenes were collected. Approximately 3–4 cm^2^ of leaf fragments were collected from each of the studied 43 populations. In total, about 390 samples were collected.

**Table 1:** Characteristics of all *Swertia perennis* populations studied.

No	Population	Country	Region	Altitude
1	EPL1	PL	Eastern Poland (Lubelska Upland)	175 11 110
2	NPL1	PL	Northern Poland (Biebrza Valley)	113
3	NPL2	PL	Northern Poland (Suwlaskie Lakeland)	110
4	NPL3	PL	Northern Poland (Suwlaskie Lakeland)	175
5	SUD1	PL	Sudetes (Karkonosze)	1310
6	SUD2	PL	Sudetes (Karkonosze)	1320
7	SUD3	PL	Sudetes (Karkonosze)	1300
8	SUD4	PL	Sudetes (Karkonosze)	1322
9	SUD5	PL	Sudetes (Karkonosze)	1287
10	SUD6	PL	Sudetes (Karkonosze)	1284
11	SUD7	PL	Sudetes (Karkonosze)	1280
12	SUD8	CZ	Sudetes (Hruby Jesenik)	1000
13	CAR1	PL	Carpathians (Beskids)	1275
14	CAR2	PL	Carpathians (Beskids)	1280
15	CAR3	PL	Carpathians (Tatry)	1017
16	CAR4	PL	Carpathians (Tatry)	851
17	CAR5	SK	Carpathians (Tatry)	1696
18	CAR6	SK	Carpathians (Welka Fatra)	580
19	CAR7	SK	Carpathians (Welka Fatra)	641
20	CAR8	SK	Carpathians (Welka Fatra)	1236
21	CAR9	SK	Carpathians (Mala Fatra)	790
22	CAR10	SK	Carpathians (Mala Fatra)	660
23	SMT1	CZ	Šumava Mountains	900
24	SMT2	CZ	Šumava Mountains	1216
25	ALP1	D	Alps (Allgäuer Hochalpen)	1122
26	ALP2	A	Alps (Allgäuer Hochalpen)	1029
27	ALP3	A	Alps (Allgäuer Hochalpen)	1124
28	ALP4	D	Schwarzwald	725
29	ALP5	CH	Alps (Glarner Alpen)	894
30	ALP6	CH	Alps (Vorapls)	1357
31	ALP7	CH	Alps (Schwyz Alpen)	1132
32	ALP8	CH	Alps (Emmentalen Alps)	1456
33	ALP9	FR	Alps (Hochalpen)	2052
34	ALP10	FR	Alps (Vanoise Alps)	2127
35	ALP11	FR	Alps (Vanoise Alps)	1707
36	JUR1	CH	Jura (Jura Vaudois)	1331
37	JUR2	FR	Jura (Jura Vaudois)	1096
38	JUR3	FR	Jura (Jura Vaudois)	1204
39	JUR4	FR	Jura (Jura Vaudois)	1100
40	PYR1	FR	Pyrenees (Catalan Pyrenees)	2032
41	PYR2	FR	Pyrenees (Catalan Pyrenees)	2140
42	PYR3	FR	Pyrenees (Catalan Pyrenees)	1606
43	PYR4	ES	Pyrenees (Huesca)	1599

*PL* Poland, *CZ* Czech Republic, *SK* Slovakia, *D* Germany, *A* Austria, *CH* Switzerland, *FR* France, *ES* Spain.

### DNA isolation, polymerase chain reaction amplification, and ISSR fingerprinting

DNA was extracted from dried plant material, using a DNeasy Plant Mini Kit (Qiagen, Hilden, Germany) according to the manufacturer’s protocol. The Inter Simple Sequence Repeat (ISSR) microsatellite markers were selected to study of *Swertia perennis* according to the method described by^25^. For the analysis, we have used primers 812, 825, 836, 841, 851, 857, and 873, which showed satisfactory amplification and generated a sufficient number of polymorphic bands representing genetic variation. The number of amplified products varied from three to 10 within a size range of 100–2000 bp, depending on the primer. PCR reactions were performed with 25 μL of sample in PCR reaction tubes, using DreamTaq DNA Polymerase (Thermo Fisher Scientific, Waltham, MA, USA). For the PCR reactions, a GeneAmp 9700 cycler (Life Technologies, Carlsbad, CA, USA) was used. Finally, the PCR ISSR amplification products were separated in agarose gel with a GeneRuler 100 bp Plus DNA Ladder (Thermo Fisher Scientific, Waltham, MA, USA) and photographed.

### Molecular data analyses

Amplification with ISSR primers was performed to obtain the DNA fingerprint profiles of *S. perennis* from all study populations. Seven primers amplified a total of 99 loci in 240 individuals from 43 European populations tested. ISSR markers after separation in agarose gel were analyzed using the CLIQS software^26^ and then corrected manually. The analysis of the fringe images allowed them to be encoded in a binary form (0,1), and the data obtained this way was used for statistical calculations. Molecular analysis of variance (AMOVA) studies were performed using the Arlequin 3.5.1 program to determine the distribution of genetic variation within and between populations, and to assess the genetic differentiation of the *S. perennis* populations studied^27^. The degree of population division was measured by the F_st_ index according to^28–30^. The effective and observed number of alleles^31^, as well as the gene diversity index by^32^, Shannon's information index^33^, and Nei’s genetic identity and genetic distance indexes^34^ were calculated using POPGENE v. 1.32^35^. The number of bands (private bands found in groups) was calculated using FAMD^36^. The STRUCTURES 2.3.4 program was used for analyses of Bayesian clustering based on the admixture model^37–39^. The split by the number of groups (K) was tested with 10 replicates per K, using 200,000 firing iterations followed by 1,000,000 MCMC iterations. The output with many K values and hundreds of iterations was analyzed using the CLUMPAK online software^40^, which produced a graphical representation of the results of Structure 2.3.4 and calculated the necessary statistics. The optimal value of K was estimated from Ln (K) and the calculation of ΔK^38^, using CLUMPAK. Coordinate analysis (PcoA) calculations and graphical visualization of data were obtained on the basis of calculations of the genetic distance matrix generated based on the obtained data, using the GeneAlEx program^41^, with the method described by^42^. To reconstruct the relationships among the analyzed populations, we used a consensus network split using the equal-angle approach as implemented in SplitsTree^43^, using 1000 bootstrap replicates. GeneAlEx with the Mantel test implemented was also used to test the relationship between the matrix of the logarithmic geographic distances and the matrix of genetic diversity values. All scientific names of plant taxa are given according to^44^. The abbreviated surnames of the authors of plant names are given as in in accordance with the recommendations of the ICN^45^. The reproducibility of SSR markers was assessed by repeated DNA extraction from eight randomly selected samples of leaves from which DNA was previously isolated. The PCR reactions were also repeated with all the primers selected for the study and the separation on agarose gel was repeated. Band analysis was also performed comparing the result with previously performed analyses. The results were identical with the approximately 3–5% difference found for the three primers.

## Results

Statistical analysis of the data showed that out of the 99 identified loci, as many as 82 were polymorphic, which proves the high allelic diversity of the individuals of the studied populations of *S. perennis* (Table 2).

**Table 2:** Analysis of genetic variation obtained with molecular markers in different *S. perennis* populations.

No	Population acronym	Na*	Ne*	H*	I*	Pl	Pb
1	EPL1	1.071	1.043	0.026 (0.017)	0.038 (0.015)	7.0	0
2	NPL1	1.061	1.036	0.022 (0.011)	0.033(0.011)	6.0	0
3	NPL2	1.061	1.036	0.022 (0.010)	0.033 (0.015)	6.0	0
4	NPL3	1.040	1.034	0.018 (0.012)	0.026 (0.026)	4.0	0
5	SUD1	1.020	1.012	0.008 (0.002)	0.011 (0.008)	2.0	0
6	SUD2	1.061	1.046	0.026 (0.011)	0.037 (0.024)	6.0	0
7	SUD3	1.010	1.018	0.010 (0.017)	0.013 (0.008)	2.0	0
8	SUD4	1.030	1.022	0.012 (0.009)	0.018 (0.007)	3.0	0
9	SUD5	1.051	1.024	0.016 (0.018)	0.025 (0.014)	5.0	0
10	SUD6	1.111	1.044	0.030 (0.015)	0.049 (0.021)	11.0	0
11	SUD7	1.061	1.020	0.015 (0.011)	0.025 (0.013)	6.0	0
12	SUD8	1.071	1.047	0.027 (0.011)	0.040 (0.008)	7.0	1
13	CAR1	1.061	1.033	0.021(0.019)	0.031 (0.022)	6.0	0
14	CAR2	1.061	1.036	0.022 (0.017)	0.032 (0.021)	6.0	0
15	CAR3	1.020	1.018	0.010 (0.011)	0.013(0.008)	2.0	0
16	CAR4	1.061	1.036	0.021 (0.011)	0.032 (0.21)	6.0	0
17	CAR5	1.272	1.103	0.071(0.008)	0.116 (0.021)	27.0	0
18	CAR6	1.131	1.064	0.040 (0.019)	0.063 (0.019)	13.0	0
19	CAR7	1.071	1.035	0.022 (0.029)	0.034 (0.015)	7.0	0
20	CAR8	1.091	1.060	0.035 (0.029)	0.052 (0.017)	9.0	0
21	CAR9	1.091	1.055	0.033 (0.028)	0.049 (0.031)	9.0	0
22	CAR10	1.061	1.026	0.017 (0.012)	0.028 (0.016)	6.0	0
23	SMT1	1.071	1.040	0.024 (0.015)	0.037 (0.022)	7.0	1
24	SMT2	1.061	1.026	0.017 (0.012)	0.028 (0.022)	6.0	0
25	ALP1	1.051	1.033	0.020 (0.014)	0.029 (0.017)	5.0	0
26	ALP2	1.061	1.043	0.024 (0.011)	0.036 (0.018)	6.0	0
27	ALP3	1.091	1.053	0.032 (0.022)	0.049 (0.014)	9.0	1
28	ALP4	1.141	1.064	0.041 (0.014)	0.064 (0.024)	14.0	0
29	ALP5	1.101	1.056	0.034 (0.016)	0.052 (0.016)	10.0	0
30	ALP6	1.051	1.024	0.016 (0.007)	0.025 (0.014)	5.0	0
31	ALP7	1.081	1.029	0.021 (0.009)	0.034 (0.025)	8.0	0
32	ALP8	1.101	1.048	0.032 (0.018)	0.049 (0.031)	10.0	0
33	ALP9	1.071	1.020	0.016 (0.009)	0.027(0.011)	7.0	0
34	ALP10	1.030	1.018	0.011 (0.001)	0.017 (0.008)	3.0	0
35	ALP11	1.121	1.044	0.031 (0.019)	0.051 (0.009)	12.0	2
36	JUR1	1.071	1.029	0.020 (0.013)	0.031 (0.002)	7.0	1
37	JUR2	1.030	1.009	0.007 (0.007)	0.010 (0.001)	3.0	1
38	JUR3	1.061	1.031	0.019 (0.031)	0.030 (0.015)	6.0	0
39	JUR4	1.061	1.030	0.020 (0.009)	0.030 (0.011)	6.0	0
40	PYR1	1.081	1.029	0.021 (0.008)	0.034 (0.006)	8.0	0
41	PYR2	1.051	1.028	0.017 (0.009)	0.026 (0.014)	5.0	0
42	PYR3	1.071	1.032	0.021 (0.011)	0.032 (0.021)	7.0	0
43	PYR4	1.182	1.057	0.043 (0.024)	0.072 (0.011)	18.0	0

Standard deviations are given in parentheses.

*Na: observed number of alleles.

*Ne: effective number of alleles^31^.

*H: gene diversity^32^.

*I: Shannon's information index^33^.

*Pl: number of polymorphic bands.

*Pb: number of private bands found in groups (only meaningful if groups are mutually exclusive).

**Figure 1:**
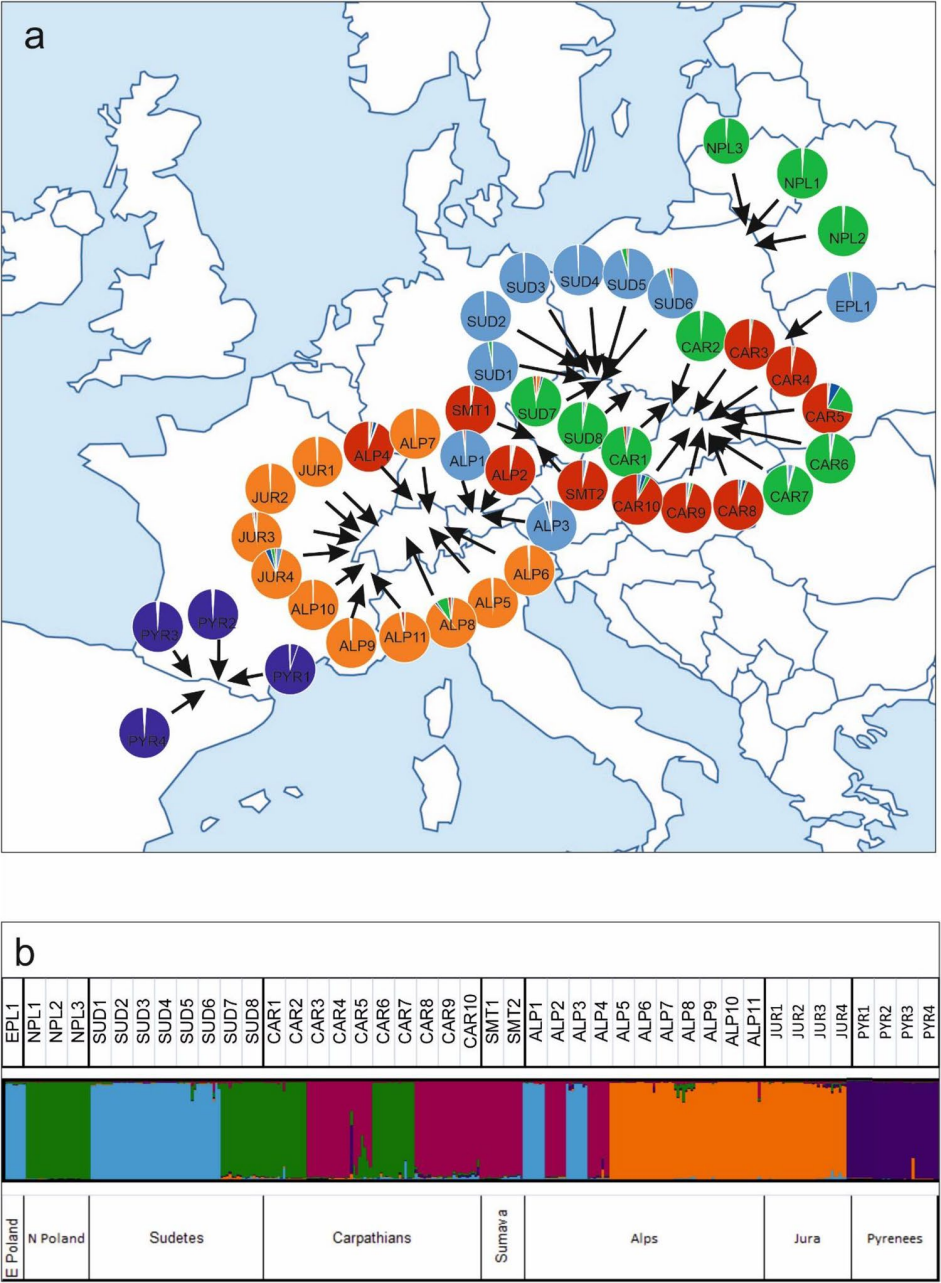
(**a**) Sample localities of *S. perennis* populations with charts describing the proportions of individuals classified into five clusters defined using the Bayesian approach. Each colour represents one of five clusters. (**b**) Direct output from STRUCTURE software results for all populations for *K* = 5.

The observed number of allels showed that Na ranged from 1.01 (SUD3) to 1.272 (CAR5). The smallest effective number of alleles (Ne and the lowest gene diversity Index, H and Shannon's Information Index—I) were found in the JUR2 population. However, the highest values of these coefficients were found in the CAR5 population. The calculated number of polymorphic bands (Pl) ranged from 2 (SUD1, SUD2) to 27 in the CAR5 population. Private bands were rarely found in *S. perennis* populations. The highest Pb (number of polymorphic bands) value (2) was found in the ALP11 population (Meribell, Alps). The genetic structure of the population was also studied by Bayesian analysis, using the STRUCTURE program. ΔK values for STRUCTURE results were estimated in the admixture analysis to assess population distribution and allele distribution between the groups (Suppl. 1). The analysis showed the highest value for ΔK = 5. Therefore, this value was used to illustrate the distribution of the population (Fig. 1a, b).

**Figure 2:**
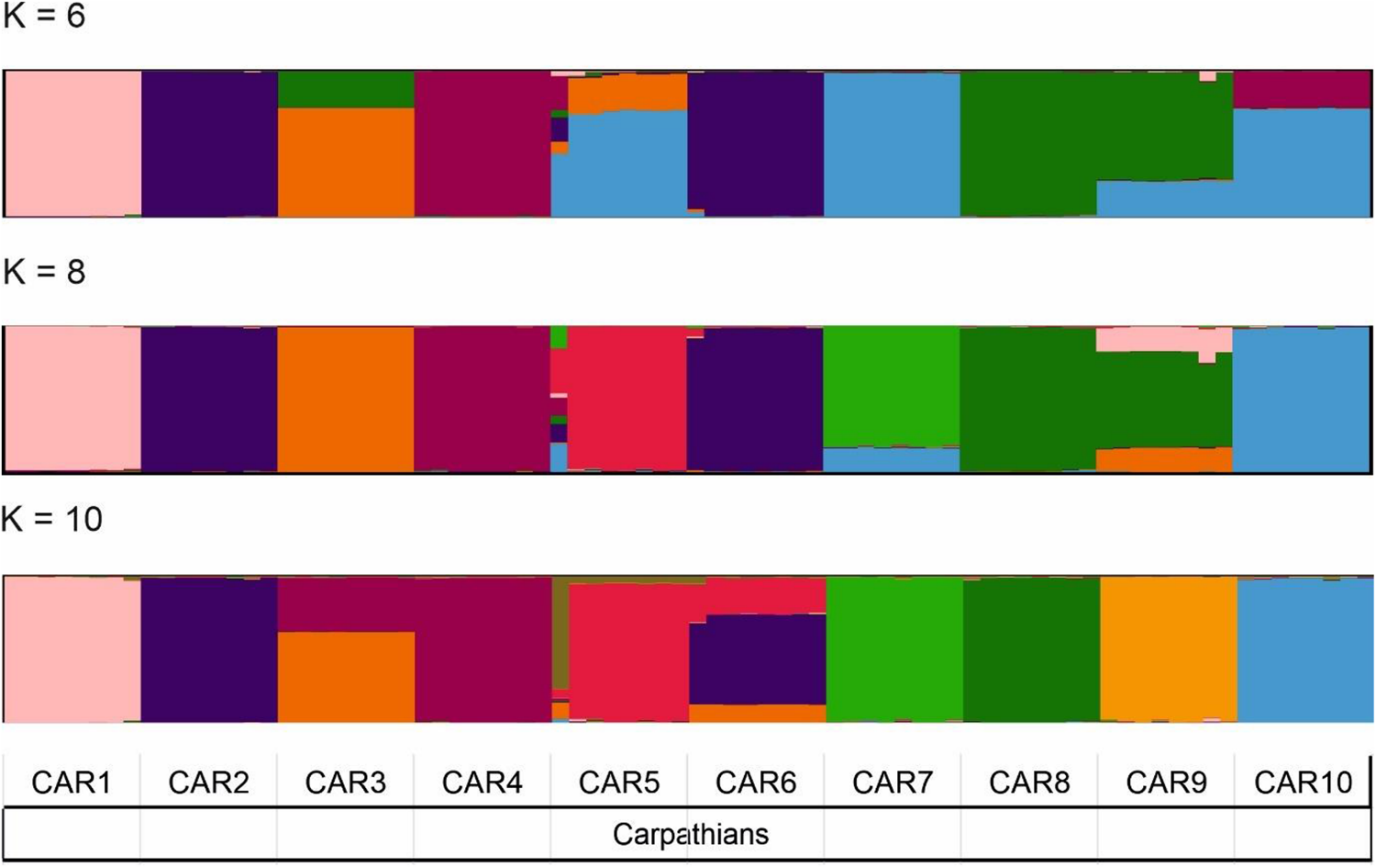
STRUCTURE software results for Carpathians populations for *K* = 6, 8 and 10.

Analysis of the STRUCTURE program the division with these values showed that, in this case, a similar allele division was maintained (K = 5) in populations from the Pyrenees, the Alps, the Sudetes, and northern and eastern Poland, whereas in the Carpathians, it was divided into many subgroups. Therefore, an additional STRUCTURE analysis was performed, based on the attempt made to determine the most probable distribution of K values by means of impurity analysis. Unfortunately, the analysis did not indicate which K value would be appropriate, and the results of the admixture suggested a very high level of genetic differentiation within the Carpathians in the K = 6–10 groups (Fig. 2). Hence, the Structure’s results revealed a clear division of the population into geographically separated groups, which indicates that the population of the Carpathians is highly distinct.

**Figure 3:**
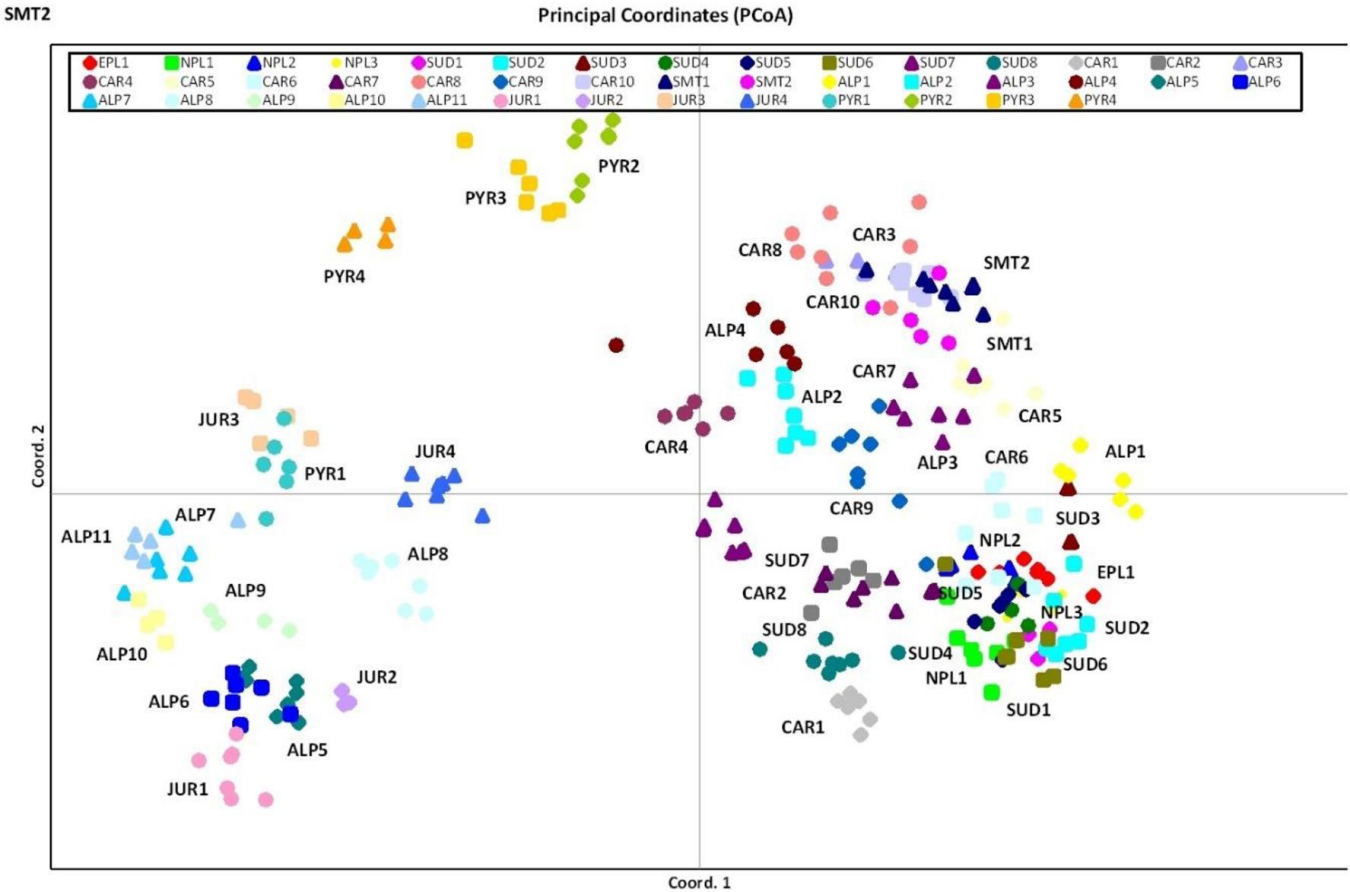
Scatter diagram of principal coordinate analysis (PCoA) *S. perennis* individuals based on ISSR data.

Genetic distinctness was evident in the populations of the Pyrenees and the Alps. In the remaining regions, gene addition was found, which may indicate a past history of gene flow between populations. PCoA based on genetic distance divided the populations into three groups: the Pyrenees population, the Alps populations, and the remaining populations from northern Poland, eastern Poland, the Carpathians, the Sudetes, and several populations from the Alps (Fig. 3).

AMOVA analyses were performed using the Arlequin program based on the predictive division of the population into six and eight groups resulting from the geographical division (Table 3).

**Table 3:** Results of ANOVA analysis in a hierarchical arrangement/with the division of the population into groups.

Source of variation	df	Sum of squares	Variance component	Variation (%)
Among all populations	42	4542.7	13.3	90.9
Within populations	301	398.6	1.32	9.02
Total	343	4941.3	14.6	
* Fst*	0.909			
Among 5 groups (Eastern and Northern Poland–Sudetes–Carpathians–Alps and Jura–Pyrenees)	4	1321.4	2.1	27.5
Among populations within groups	16	3290.2	11.1	62.5
Within populations	301	361.2	2.54	7.54
Total	343	5941.3	14.7	
* Fst*	0.834			

**Figure 4:**
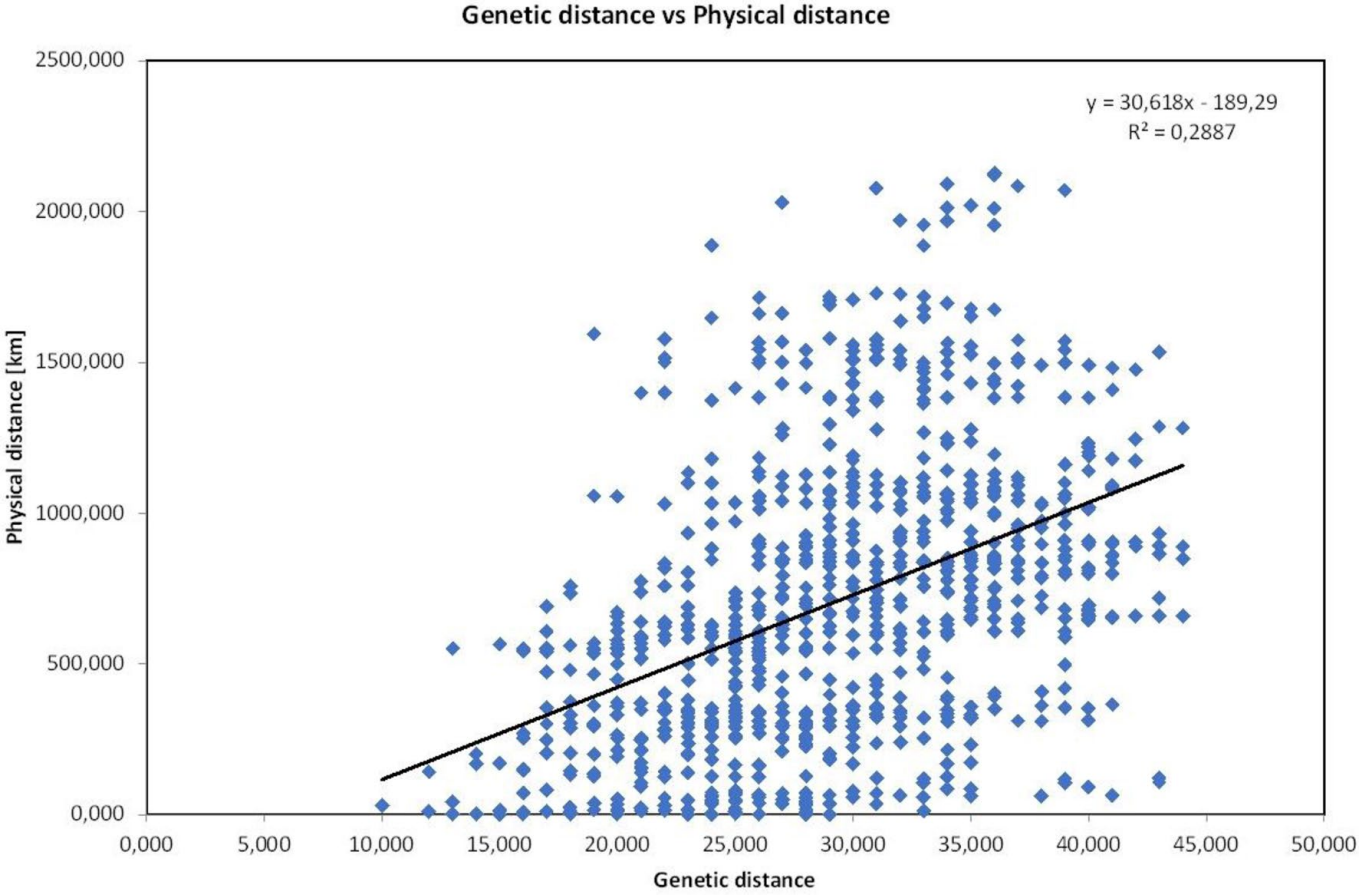
The correlation between Nei (1973) genetic distance and logarithm of pairwise geographical distance between populations of *S. perennis.*

Only 8.64% to 9.02% of the total variability found was due to differences between individuals within the population. Variation among populations within groups ranged from 71.4 to 72.3%, which indicates high variability between the studied populations. A very high and statistically significant (*p* < 0.01) F_st_ index was also found, which reflected the variability among the studied populations of *S. perennis*. AMOVA was high for all populations (F_st_ = 0.909) and for population groups (F_st_ = 0.834). This was also confirmed by the results obtained for the analyses of the genetic distance matrix of all populations (Suppl. 2). The results of the Mantel test indicate a high and significant positive relationship between the geographical distances of the *S. perennis* populations and the genetic distance (R = 0.288; *p* < 0.01) in all randomized locations, which indicates isolation by distance (Fig. 4).

**Figure 5:**
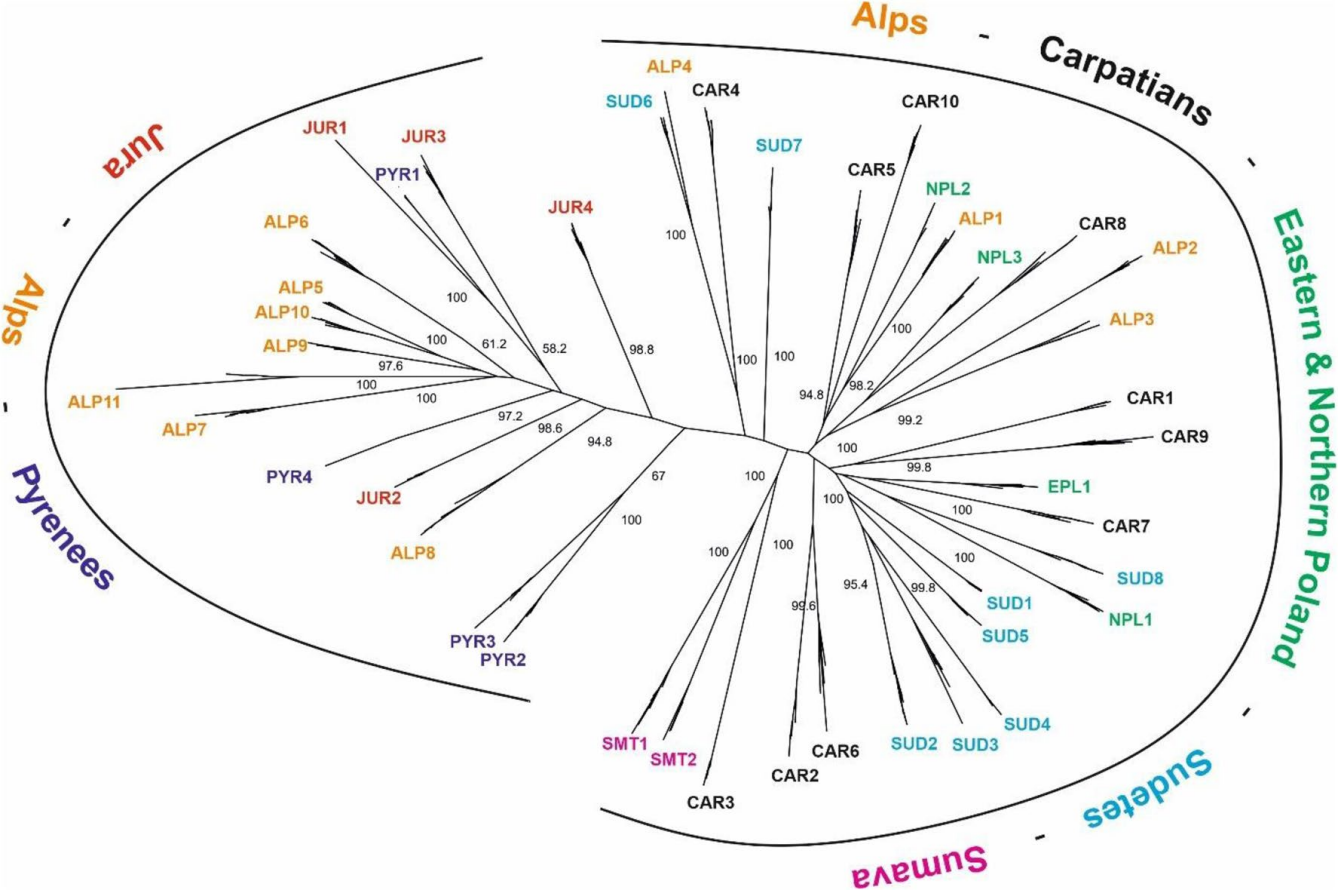
Results of SplitsTree neighbour—net clustering of studied populations *S. perennis*.

The result of the SplitsTree analysis points to the division of the *S. perennis* metapopulation in Europe into two groups. The western group, which includes populations from the Pyrenees, the Jura Mountains, and the Alps, and the eastern group, with populations from northern and eastern Poland, the Carpathians, the Sudetes, and Šumava Mountains, with an admixture of Alpine populations (Fig. 5). Differences between the populations of *S. perennis* from the Alps are also visible, which are not homogeneous in the area from which the samples for research were taken. There are visible differences between the samples from the Central Alps (ALP 1–4, ALP7) collected in western Austria and eastern Switzerland, and between the samples collected from the Jura and the western Alps. Some Central Alpine populations show similarities to the Western Carpathians and the Šumava Mountains, and some to the Sudetes.

## Discussion

Genetic differentiation among *S. perennis* populations is very high (F_st_ = 0.909) compared to other plant species like *Trollius europaeus* L., F_st_ = 0.39; *Cicerbita alpina*, F_st_ = 0.30; *Ranunculus platanifolius*, F_st_ = 0.30 or *Lonicera nigra*, F_st_ = 0.37^46–49^ in a similar geographical area. The found high value of the F_st_ coefficient seems quite high. However, the previously cited results of these coefficients were calculated for data obtained using the AFLP method, which, although they concern other species of herbaceous plants, but the set of samples for testing was made in similar locations. Both methods are obviously not comparable in the case of F_st_ calculations, but the obtained values can be classified as small, medium or large. A similar value for AFLP was calculated for the population of *Hieracium* sect. *Cernua* (Fst = 0.99), and in the case of the ISSR method 0.47^50^ for Kirengeshoma and F_st_ = 0.42 for *Bromus erectus*^51^. It can be definitely said that the result obtained by us is of high value and clearly indicates a large variation between *Swertia perennis* populations.

Based on the results of STRUCTURE analyses (Figs. 1a, b, 2 a, b), PcoA analyses (Fig. 3), and nearest neighbor analyses (Fig. 4), at least five different biogeographical groups of *S. perennis* can be identified (represented by populations from the Pyrenees, the Alps, the Sudetes, the Carpathians, and northern Poland). Analyses of the Pyrenees population revealed a substantial discrepancy with other populations. Similar results were reported for the genetic variability of other plant species^8,52,53^, indicating a large diversity in the Pyrenean populations of different species. Nonetheless, the studied populations (including *S. perennis*) were not internally differentiated. This may be due to the Würm glaciation, which lasted from 115,000 to about 11,700 years ago and which probably affected the genetic disjunction of plant and animal populations. Despite the presence of local glaciations, the Pyrenees are thought to have been one of the Pleistocene glacial refugia, a potential refuge area for many Arctic-Alpine and mountain species^54,55^. Another reason could be the bottleneck effect, which is often the result of a sudden decline in numbers caused by environmental events caused by a sudden random event, e.g. a catastrophe. This results in a reduction in genetic variability (reduced genetic diversity in the population). In this case, genetic diversity can only increase through gene flow from another population, which was not the case with the population from the Pyrenees. That is why the studied populations from the Pyrenees show such a large genetic distinctness. It is also possible that these geographically isolated populations are descended from a limited number of individuals who established them. Thus, the occurrence of the founder effect and the related occurrence of genetic drift can also be considered as equally likely for the population from the Pyrenees^56,57^.

This is confirmed by the results obtained by the PcoA analyses, which indicate the existence of three large differences between the populations of the Alps and the Pyrenees, but also between the populations of the Alps and those of other geographical regions (the Carpathians, southern Bohemia, the Sudetes and northern Poland). Population groups from the Alps, including those located in the Jura Mountains, the Western Alps, and the Central Alps, are poorly differentiated internally. However, the populations of the Alps (ALP1, ALP2, ALP3, and ALP4) differ from others and show some similarities to the populations of the Carpathians and the Sudetes (Fig. 1a, b). It is also interesting that one of the northernmost populations from Germany (ALP4) is colored red (Fig. 1a). This population comes from the Schwarzwald and, like the populations from north-eastern Poland (green) and southern Poland (blue), is a relic of the former wider distribution of these (periglacial) genetic groups within Swertia perennis.

This third genetic group is the populations of the Eastern Alps, Sudetes (blue), Carpathians and Poland. Lowland populations are characterized by low genetic diversity within populations, but high genetic diversity between populations. This pattern has previously been attributed to relict populations^58,59^ and may be due to a reduction in population size with limited gene flow leading to strong genetic drift after the expansion of forests in the Holocene. In the case of *S. perennis*, this process could also occur in lowland populations, which are relict in nature, confirming the presence of unique alleles in some populations. Lowland populations are therefore probably the result of two processes—postglacial colonization from glacial refugia and interglacial contraction of relict lowland populations. In special cases, the source of post-glacial colonization of northern areas could have been populations from the Western Carpathians or the Eastern Alps^59,60^. At that time, the possibility of settling the areas of northern Poland arose, but also a slow migration of various species to the south, probably along with migrating plant communities. The conditions created in the periglacial zone and the system of wet river valleys with numerous peatland areas, which could have been inhabited by *S. perennis* populations, were certainly conducive to this. This is another plausible explanation for the large similarity between the populations from northern Poland and those from the Sudetes and the Carpathians (Fig. 1a, b). Within this third group, the STRUCTURE analysis identified three (sub)clusters that are mixed and do not follow a simple geographic pattern. There is a visible tendency to group populations from the Sudetes with the Eastern Alps and from southern Poland. The two visible clusters with the Carpathians are associated with the northernmost populations of Poland (green), which are geographically close. There are also visible connections between the Carpathian population and the Eastern Alps and the Sumava Moutains (red). The relationships between the populations of these mountain ranges are well documented. In the case of *Carex bigelowii*, several gene exchanges took place between the Alps and the Carpathians^8,21^. This may indicate frequent contact between the Sudetes, the Alps and the Carpathians with periglacial populations of various plant species, including *S. perennis* south of the ice sheet. This indicate on a wider contact of the Sudetes population with large periglacial populations south of the ice sheet, which was favored by the northernmost location of the Sudetes. This may have helped Alpine and other plant groups survive in situ in deep valleys. Hence, the Carpathians are often mentioned as refuges for many cold-adapted species^21,61,62^.

The results also suggest that the post-glacial history of the range of this species in Europe was influenced by glacial refugia of populations located both in the Alps and in the area associated with Central Europe, resulting in a visible gene addition. On the other hand, the high distinctiveness of the *S. perennis* populations from the Carpathians supports the hypothesis of a different population history for the Carpathians as the main center of mountain flora in Central Europe. Gene exchange between these population groups may have occurred in the Hercynian mountain ranges. The results also suggest that the post-glacial range history of this species in Europe was influenced by glacial refugia of populations located both in the Alps and in the area associated with Central Europe, resulting in a visible addition of genes^21^. On the other hand, the high distinctness of the population of S. perennis from the Carpathians supports the hypothesis of a different population history of the Carpathians as the main center of mountain flora in Central Europe. As in the case of *Ranunculus platanifolius*^47^, the populations of *S. perennis* from the Carpathians show similarities to the populations from the Sudetes.

The area of the Carpathians is not very extensive, but it is physiographically diverse and is crossed by numerous mountain ranges, which may be the habitat of genetically diverse populations of *S. perennis*. This explains the isolation of the current localities of *S. perennis* in the Carpathians (Fig. 2). Carpathian population seems to be genetically isolated, as indicated by the number of clusters identified in the STRUCTURE analysis (Fig. 2). Distinguishing geographically close populations in separate, near-pure clusters supports the notion of very limited gene flow between these populations. Such a clear genetic differentiation of closely located populations is rarely found. Such a picture of the genetic diferntiation of the Carpathian populations suggests that these populations survived in this area, being numerous enough to maintain the observed level of genetic variability. The result of the STRUCTURE study may also suggest the presence of a founder effect in those populations of *S. perennis* that are geographically isolated from each other. Carpathians were only locally glaciated, and the snow line was estimated at 1700–1800 m a.s.l. Below this level, siliceous substrates provided suitable habitats for Alpine plants during the glacial period^9^. The Carpathian species probably did not experience a regional extinction as in the Alps, but instead, they may have covered larger areas than today with intensified gene flows, creating a large gene pool among formerly distant populations^63^. Similar mechanisms have also been proposed for other Alpine plants endemic to the Eastern Alps^64^. Perhaps, local cryptic refugia played a role in the Carpathians. Such refugia are particularly important for non-Arctic species in Central Europe, with isolated localities in their foreland (periglacial areas) and in lowland areas apart from mountains^52,57,65–67^. Thus, the existing populations of *S. perennis* in the Western Carpathians probably survived the glacial periods in many refugia. Also in the populations of *Cicerbita alpina* and *Ranunculus platanifolius* in deep valleys in the southern part of the Western Carpathians, a different genetic pattern was found^46,47^. Differences in the genetic structure of plant populations in the Carpathians are reflected in the presence of separate phylogroups, but also in their internal heterogeneity. This indicate on greater isolation of local Western Carpathian populations and/or the possibility of secondary contacts.

In conclusion, the history of *S. perennis* post-glacial migrations are complicated, especially in Central Europe, where gene exchange and close phylogenetic relationships can be observed in some mountain ranges. Our research also confirms that both the Carpathians and the Sudetes as well as populations from Polish lowlands and in southern Germany were the refuges where gene admixture took place. The obtained results suggest that these sites played a crucial role in the overall survival of *S. perennis*. Certainly, there was gene exchange between populations located in the eastern Alps, the Carpathians and the Hercynian mountain ranges (Bohemian Forest, Jeseniky, Sudetes, Erzgebirge). This confirms earlier results of comparative studies on genetic differentiation of populations of other vascular plant species^21,46^. However, for more decisive conclusions, synthetic studies that are based not only on molecular markers but also on paleobotanical studies and population dispersion at a distance are needed.

### Supplementary Information

Supplementary Information 1.

Supplementary Information 2.

## Data Availability

All of the data used for the analyses are available for Editors, Reviewers and Redaers on request. Please contact with corresponding Author.
