# Supplementary material for: The molecular population structure of Swertia perennis (Gentianaceae) in Central Europe
Source: Sci Rep. 2023 Oct 10;13:17059. doi: 10.1038/s41598-023-43731-5 (PMC10564900; doi:10.1038/s41598-023-43731-5)
Supplement: Supplementary file 1 — Supplementary Information 1. [file 41598_2023_43731_MOESM1_ESM.pdf]

Suppl 1. Genetic distance of studied *S. perennis* populations.

	EPL1	NPL1	NPL2	NPL3	SUD1	SUD2	SUD3	SUD4
EPL1	0,000							
NPL1	0,874	0,000						
NPL2	0,882	0,867	0,000					
NPL3	0,838	0,892	0,871	0,000				
SUD1	0,790	0,901	0,891	0,873	0,000			
SUD2	0,884	0,934	0,929	0,932	0,850	0,000		
SUD3	0,898	0,937	0,928	0,926	0,884	0,933	0,000	
SUD4	0,873	0,904	0,902	0,905	0,848	0,925	0,905	0,000
SUD5	0,812	0,894	0,875	0,846	0,801	0,867	0,855	0,855
SUD6	0,916	0,925	0,902	0,918	0,922	0,946	0,947	0,919
SUD7	0,846	0,890	0,898	0,893	0,864	0,927	0,916	0,875
SUD8	0,878	0,933	0,938	0,917	0,876	0,946	0,939	0,910
CAR1	0,876	0,897	0,897	0,877	0,887	0,942	0,937	0,912
CAR2	0,883	0,912	0,886	0,896	0,893	0,934	0,933	0,897
CAR3	0,895	0,931	0,927	0,905	0,918	0,941	0,945	0,931
CAR4	0,777	0,764	0,737	0,808	0,800	0,827	0,838	0,794
CAR5	0,803	0,807	0,825	0,836	0,831	0,852	0,882	0,847
CAR6	0,873	0,897	0,881	0,871	0,887	0,933	0,920	0,912
CAR7	0,864	0,877	0,880	0,902	0,867	0,909	0,901	0,878
CAR8	0,860	0,903	0,894	0,879	0,877	0,926	0,896	0,882
CAR9	0,910	0,900	0,916	0,931	0,909	0,937	0,944	0,919
CAR10	0,921	0,941	0,933	0,933	0,938	0,952	0,959	0,955
SMT1	0,919	0,925	0,919	0,915	0,913	0,933	0,926	0,908
SMT2	0,898	0,900	0,883	0,882	0,906	0,931	0,929	0,917
ALP1	0,894	0,929	0,914	0,913	0,897	0,927	0,935	0,930
ALP2	0,830	0,893	0,884	0,889	0,843	0,904	0,898	0,861
ALP3	0,873	0,903	0,883	0,882	0,887	0,910	0,899	0,877
ALP4	0,914	0,917	0,908	0,921	0,917	0,930	0,927	0,915
ALP5	0,939	0,947	0,943	0,951	0,938	0,961	0,959	0,948
ALP6	0,938	0,949	0,943	0,940	0,938	0,960	0,955	0,949
ALP7	0,889	0,908	0,898	0,890	0,900	0,927	0,933	0,915
ALP8	0,930	0,952	0,940	0,943	0,929	0,960	0,956	0,946
ALP9	0,938	0,957	0,949	0,943	0,943	0,971	0,966	0,956
ALP10	0,923	0,936	0,925	0,930	0,922	0,946	0,939	0,931
ALP11	0,925	0,937	0,937	0,930	0,929	0,960	0,946	0,935
JUR1	0,942	0,954	0,947	0,936	0,949	0,976	0,971	0,959
JUR2	0,935	0,949	0,935	0,934	0,938	0,961	0,951	0,942
JUR3	0,897	0,936	0,914	0,918	0,907	0,942	0,947	0,928
JUR4	0,875	0,912	0,897	0,910	0,847	0,907	0,907	0,880
PYR1	0,902	0,942	0,932	0,935	0,916	0,952	0,952	0,936
PYR2	0,904	0,939	0,929	0,922	0,915	0,945	0,942	0,925
PYR3	0,894	0,905	0,901	0,906	0,897	0,914	0,922	0,900
PYR4	0,923	0,939	0,930	0,933	0,932	0,958	0,950	0,937

SUD5	SUD6	SUD7	SUD8	CAR1	CAR2	CAR3	CAR4	CAR5
0,000								
0,903	0,000							
0,879	0,896	0,000						
0,893	0,944	0,908	0,000					
0,877	0,919	0,897	0,913	0,000				
0,842	0,930	0,894	0,934	0,898	0,000			
0,888	0,933	0,909	0,946	0,927	0,923	0,000		
0,804	0,814	0,797	0,823	0,797	0,788	0,814	0,000	
0,821	0,849	0,864	0,850	0,789	0,865	0,896	0,670	0,000
0,888	0,923	0,863	0,905	0,905	0,907	0,914	0,772	0,856
0,873	0,906	0,883	0,906	0,889	0,883	0,900	0,748	0,852
0,855	0,894	0,872	0,900	0,889	0,876	0,897	0,760	0,832
0,911	0,936	0,914	0,939	0,920	0,931	0,929	0,792	0,874
0,920	0,952	0,939	0,969	0,936	0,938	0,932	0,841	0,889
0,895	0,930	0,926	0,934	0,926	0,933	0,945	0,813	0,862
0,889	0,914	0,906	0,936	0,900	0,916	0,912	0,747	0,856
0,882	0,925	0,915	0,937	0,912	0,921	0,915	0,798	0,838
0,863	0,909	0,853	0,910	0,891	0,902	0,910	0,739	0,827
0,864	0,873	0,875	0,910	0,897	0,889	0,870	0,798	0,849
0,895	0,900	0,905	0,937	0,904	0,896	0,911	0,847	0,881
0,931	0,934	0,923	0,964	0,942	0,937	0,950	0,876	0,913
0,929	0,936	0,928	0,957	0,926	0,939	0,924	0,864	0,912
0,901	0,896	0,887	0,923	0,894	0,891	0,894	0,808	0,867
0,922	0,948	0,919	0,970	0,941	0,935	0,928	0,866	0,914
0,941	0,949	0,926	0,971	0,945	0,946	0,943	0,866	0,920
0,915	0,922	0,912	0,945	0,924	0,916	0,917	0,853	0,897
0,916	0,933	0,901	0,952	0,920	0,923	0,923	0,868	0,907
0,938	0,955	0,941	0,972	0,937	0,947	0,947	0,865	0,905
0,923	0,933	0,921	0,958	0,933	0,937	0,917	0,837	0,910
0,900	0,929	0,918	0,955	0,925	0,912	0,930	0,825	0,881
0,866	0,930	0,891	0,913	0,917	0,911	0,919	0,751	0,817
0,910	0,943	0,918	0,954	0,918	0,933	0,932	0,833	0,891
0,912	0,924	0,903	0,944	0,919	0,922	0,929	0,823	0,890
0,882	0,857	0,878	0,928	0,894	0,901	0,864	0,806	0,860
0,923	0,923	0,925	0,949	0,916	0,929	0,937	0,847	0,891

CAR6	CAR7	CAR8	CAR9	CAR10	SMT1	SMT2	ALP1	ALP2
0,000								
0,872	0,000							
0,875	0,843	0,000						
0,916	0,894	0,910	0,000					
0,940	0,910	0,909	0,946	0,000				
0,921	0,889	0,910	0,921	0,942	0,000			
0,899	0,848	0,898	0,919	0,932	0,884	0,000		
0,917	0,896	0,873	0,934	0,940	0,924	0,890	0,000	
0,889	0,854	0,871	0,892	0,936	0,900	0,889	0,870	0,000
0,903	0,867	0,876	0,894	0,907	0,883	0,892	0,893	0,835
0,908	0,899	0,905	0,924	0,931	0,914	0,915	0,900	0,905
0,942	0,920	0,927	0,948	0,964	0,951	0,948	0,937	0,930
0,931	0,921	0,924	0,948	0,961	0,941	0,936	0,923	0,924
0,892	0,877	0,887	0,922	0,928	0,920	0,902	0,909	0,894
0,936	0,919	0,923	0,953	0,958	0,954	0,945	0,930	0,916
0,940	0,929	0,929	0,958	0,972	0,959	0,950	0,934	0,922
0,919	0,894	0,905	0,932	0,945	0,932	0,930	0,921	0,912
0,926	0,916	0,916	0,942	0,955	0,947	0,937	0,933	0,923
0,941	0,927	0,931	0,966	0,974	0,957	0,947	0,947	0,937
0,931	0,902	0,907	0,948	0,955	0,940	0,930	0,922	0,917
0,919	0,882	0,914	0,943	0,950	0,932	0,925	0,924	0,884
0,906	0,822	0,875	0,920	0,948	0,912	0,910	0,895	0,825
0,928	0,868	0,910	0,926	0,943	0,939	0,932	0,932	0,900
0,917	0,882	0,891	0,912	0,942	0,921	0,920	0,924	0,885
0,901	0,867	0,881	0,895	0,901	0,901	0,887	0,877	0,886
0,929	0,897	0,903	0,941	0,950	0,938	0,937	0,933	0,918

ALP3	ALP4	ALP5	ALP6	ALP7	ALP8	ALP9	ALP10	ALP11
0,000								
0,879	0,000							
0,923	0,792	0,000						
0,898	0,844	0,909	0,000					
0,869	0,849	0,877	0,853	0,000				
0,911	0,856	0,915	0,908	0,888	0,000			
0,914	0,876	0,918	0,886	0,857	0,854	0,000		
0,886	0,867	0,865	0,865	0,851	0,863	0,866	0,000	
0,904	0,838	0,889	0,888	0,871	0,868	0,863	0,850	0,000
0,914	0,892	0,952	0,920	0,871	0,942	0,939	0,910	0,883
0,898	0,892	0,927	0,896	0,891	0,908	0,906	0,855	0,895
0,894	0,874	0,919	0,922	0,863	0,868	0,904	0,876	0,913
0,858	0,929	0,953	0,947	0,918	0,945	0,954	0,926	0,944
0,889	0,915	0,950	0,944	0,910	0,931	0,939	0,918	0,927
0,878	0,896	0,925	0,921	0,873	0,928	0,919	0,906	0,922
0,842	0,835	0,875	0,857	0,847	0,867	0,881	0,847	0,865
0,908	0,880	0,906	0,914	0,856	0,914	0,897	0,842	0,886

JUR1	JUR2	JUR3	JUR4	PYR1	PYR2	PYR3	PYR4
0,000							
0,928	0,000						
0,919	0,905	0,000					
0,957	0,935	0,929	0,000				
0,953	0,933	0,903	0,933	0,000			
0,948	0,921	0,902	0,934	0,855	0,000		
0,896	0,839	0,860	0,911	0,861	0,836	0,000	
0,923	0,884	0,886	0,941	0,896	0,902	0,876	0,000
