# Supplementary figures and images for: The molecular population structure of Swertia perennis (Gentianaceae) in Central Europe
Source: Sci Rep. 2023 Oct 10;13:17059. doi: 10.1038/s41598-023-43731-5 (PMC10564900; doi:10.1038/s41598-023-43731-5)

## Suppl 2. Analysis of the best K ratio from STRUCTURE software

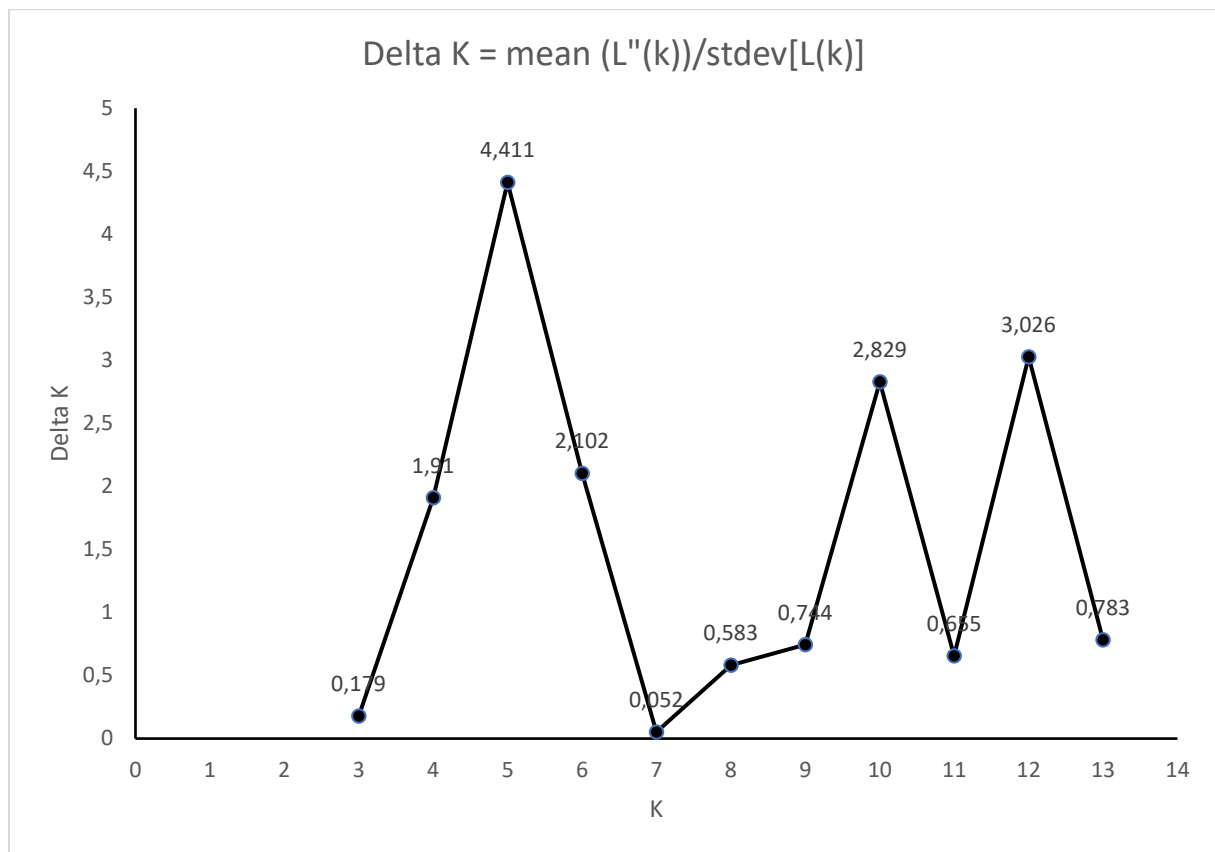

Supplement: Supplementary file 2 — Supplementary Information 2. [file 41598_2023_43731_MOESM2_ESM.pdf]
